# Responsiveness of the neurodisability scale for meningoencephalitis of unknown origin in dogs

**DOI:** 10.1111/jsap.13889

**Published:** 2025-06-04

**Authors:** R. Gonçalves, T. W. Maddox, S. Phillipps, J. C. Carrete, F. E. Anderson, R. T. Bentley, G. Walmsley

**Affiliations:** ^1^ Department of Small Animal Clinical Science University of Liverpool Neston UK

## Abstract

**Objectives:**

To determine the responsiveness of the neurodisability scale during the treatment of meningoencephalitis of unknown origin in dogs.

**Materials and Methods:**

The neurodisability scale score was determined at initial presentation and then repeated at each reassessment during treatment. At each visit, a subjective clinical evaluation of the response to treatment was also recorded. Responsiveness of the neurodisability scale between timepoints was evaluated using the receiver operating characteristics method and correlation analysis. Responsiveness was calculated between the neurodisability scale score at initial assessment and the first re‐examination after starting treatment (*T*_1_). It was also calculated between the neurodisability scale score at *T*_1_ and a second reassessment (*T*_2_) where the score had changed either due to relapse or further improvement (if no changes occurred, the last available assessment was used).

**Results:**

Thirty‐eight dogs were included. Median time between *T*_0_ and *T*_1_ was 3 weeks, and 35/38 had shown clinical improvement. Median time between *T*_1_ and *T*_2_ was 6 months; 13 dogs were suspected to have clinical relapse. The neurodisability scale demonstrated excellent responsiveness at both timepoints, with area under the curves of 0.96 (95% CI = 0.89 to 1) at *T*_1_ and 0.93 (95% CI = 0.85 to 1) at *T*_2_. There was also an excellent negative correlation coefficient produced by the change in score and the dogs’ subjective clinical evaluation (*T*_0_ − *T*_1_ Gamma = −0.8 and *T*_1_ − *T*_2_ Gamma = −0.88).

**Clinical Significance:**

The neurodisability scale is a responsive monitoring tool during meningoencephalitis of unknown origin treatment and relapse. Our results support the utility of the neurodisability scale as a clinician‐reported outcome measure for use in clinical trials.

## INTRODUCTION

Canine meningoencephalitis of unknown origin (MUO) is a debilitating disease and despite the initiation of appropriate treatment, 25% to 33% of dogs die within one week of diagnosis (Cornelis et al., 2016; Lowrie et al., 2013). Reported longer term outcomes show that 69% of dogs are alive at 3 months (Lawn & Harcourt‐Brown, 2022), 64% at 6 months (Gonçalves et al., 2024b) and 48% at 12 months (Gonçalves et al., 2024a) after diagnosis. The term MUO includes the subtypes, granulomatous meningoencephalomyelitis, necrotising meningoencephalitis and necrotising leucoencephalitis, which can only be distinguished through histopathology (Cornelis et al., 2019). The treatment for MUO currently relies on immunosuppression through the use of corticosteroids (Jeffery & Granger, 2023). This is often complemented by other medications, such as cytosine arabinoside, ciclosporin, lomustine, azathioprine, procarbazine, leflunomide and mycophenolate mofetil (Cornelis et al., 2019). At this time, large prospective, blinded randomised clinical trials comparing the different treatments directly are not available, and treatment protocols are mostly directed by owner and clinician preference. Such clinical trials are essential to identify the best treatment for MUO, but importantly, the development and use of robust outcome measures will be crucial to estimate a patient’s degree of disability at any point during the trial, and to assess changes that might be attributable to treatment effects.

The neurodisability scale (NDS) has been recently reported as a proposed outcome measure for canine MUO (Gonçalves et al., 2023). The NDS is a 21‐point scale, which attributes a numerical rating of dysfunction (0 to 3) in seven categories. The NDS was developed to try to overcome the inherent difficulties of using survival as an outcome measure in veterinary medicine. In companion animal species, survival is often affected by external factors such as owner financial limitations and the concept of acceptable quality of life, which can result in euthanasia independent of response to treatment. A higher NDS score at the time of diagnosis has been associated with increased risk of death within 6 months after diagnosis, relapse and incomplete resolution of the clinical signs within that time (Gonçalves et al., 2024b) as well as with lesion load on Magnetic Resonance Imaging (MRI) (Gonçalves et al., 2024a).

In the development of a clinical outcome measure (COM), thorough evaluation of a scale’s psychometric properties is imperative. A useful COM should be valid, reliable and responsive (Boateng et al., 2018; Inojosa et al., 2020; Narayanaswami, 2017). The NDS showed high interobserver reliability (degree of consistency exhibited when a measurement is repeated under identical conditions) for the total NDS score, but validity and responsiveness could not be assessed in the previous study (Gonçalves et al., 2023). Validity represents the extent to which an instrument measures what it was developed to evaluate (Boateng et al., 2018; Kimberlin & Winterstein, 2008; Narayanaswami, 2017). Due to the lack of similar outcome measures for MUO, this remains difficult to assess at this time. Responsiveness measures an instrument’s ability to capture change (Boateng et al., 2018; Kimberlin & Winterstein, 2008; Narayanaswami, 2017). As clinicians are most commonly interested in evaluating change in function over time in patients with chronic disease (such as MUO), in clinical trials, outcome measures with high sensitivity to change would be preferable. Reliability and validity of COMs are most important for descriptive or predictive purposes, whilst responsiveness is the essential attribute that determines whether a COM is suitable to use in a clinical trial or other longitudinal evaluations (Narayanaswami, 2017).

Methods for measuring responsiveness are complex. Longitudinal studies are needed to determine whether an instrument is responsive to changes or differences. These studies include clinical trials comparing treatments of known efficacy or observational studies where patients are treated with standard medical care and followed over relevant periods of time (Revicki et al., 2008). To assess responsiveness, criteria that allow identification of patient change (either improved or deteriorated) over time is necessary. These criteria, or anchors, may be clinician‐reported outcome measures or some combination of clinical and patient‐based outcomes. The main types of COMs include clinician‐reported, patient‐reported, observer‐reported and performance outcome measures (Narayanaswami, 2017). Although in human medicine, patient‐reported outcome measures are often prioritised as they focus on the effectiveness of the intervention from the patient’s perspective, these cannot be used in veterinary medicine, so clinician‐reported outcome measures seem most appropriate. These are assessments that are performed by investigators with appropriate professional training related to the measurement and interpretation of the outcome and typically involve interpretation of observable manifestations related to the condition of interest. The anchor‐based approach to responsiveness determination assigns patients to subgroups based on the degree of change (none, small and large); specifically, the change in the score given by the COM is compared with external evidence of change (real change).

The aim of this study was therefore to investigate the responsiveness of the NDS in a population of dogs recently diagnosed with MUO after initiation of treatment in order to determine whether it would be an appropriate COM for future clinical trials in this condition.

## MATERIALS AND METHODS

Dogs diagnosed with MUO at the Small Animal Teaching Hospital (SATH) of the University of Liverpool were prospectively enrolled. Ethics approval for this study was granted by the Ethics Committee of the University of Liverpool (VREC1229). Dogs were presumptively diagnosed with MUO if the following criteria were satisfied: (a) older than 6 months of age; (b) multiple, single or diffuse intra‐axial hyperintensities on *T*_2_‐weighted MR images; (c) mononuclear pleocytosis on Cerebrospinal fluid (CSF) analysis (dogs were not excluded if CSF analysis was not performed due to signs of raised intracranial pressure on MRI) and (d) negative titres for *Toxoplasma gondii* and *Neospora caninum* (Granger et al., 2010). Each dog was examined on initial and subsequent presentation by a board‐certified veterinary neurologist and/or a supervised veterinary neurology resident and underwent a complete neurological examination including gait analysis, postural reaction testing, cranial nerve examination, spinal reflex assessment and paraspinal palpation. The NDS standard sheet was then completed, and the total NDS score was calculated. To be included, dogs had to have at least two NDS scores: one at the time of diagnosis and the second a few weeks after starting appropriate treatment; dogs that died before re‐examination were therefore excluded from the study.

The purpose of a responsiveness study was to evaluate the measurement property of a given outcome measure and not the effectiveness of therapy (Narayanaswami, 2017); therefore, we did not rigidly control the treatment for each patient, as this is often guided by clinician and pet owner preference (often related to financial considerations and time availability to return for veterinary hospital appointments). Nonetheless, all patients received immunosuppressive doses of prednisolone and an 8‐hour constant rate infusion of cytosine arabinoside (200 mg/m^2^) immediately after diagnosis. Use of other long‐term medications was recorded.

The NDS was repeated at each reassessment at the veterinary hospital after initiation and during treatment. Patients were reassessed at 3 to 4 weeks after initial diagnosis and then at different timepoints depending on progress. At each assessment, a subjective clinical evaluation of the response to treatment (the external criterion of change) was determined by the primary clinician in charge of the case. This was calculated by considering the clinical history, owner’s perception of the animal’s progress and quality of life and the findings of the physical and neurological examinations. Based on these findings, the response to treatment was categorised as markedly worse, slightly worse, no change, slightly better or markedly better. In some cases, the assessor of the NDS score was the same assessor of the subjective score, but the latter was not only based on the findings of the neurological examination and took many other variables into account.

### Statistical analysis

Data were analysed using Microsoft Excel and SPSS, Version 28 (SPSS Inc., Chicago, IL). Continuous data were assessed for normality using the Shapiro–Wilk test. Descriptive statistics are reported for continuous variables using mean (standard deviation; SD) for approximately normally distributed variables, median (interquartile range; IQR) for variables with skewed distributions and frequencies [with 95% confidence intervals (CI) where appropriate] for categorical variables.

In this study, the responsiveness was evaluated using the receiver operating characteristics [ROC, 95% confidence interval (CI)] method and correlation analysis. These responsiveness indices have been commonly used previously to assess the responsiveness of outcome measures following intervention in different populations (De Vet et al., 2001; Lehman & Velozo, 2010). The ROC curve summarises the ability of a measure to distinguish between improved and unimproved subjects by plotting the hit (sensitivity) and false alarm (1‐specifity) rates for a series of incremental change scores in that measure. Sensitivity was defined as the proportion of patients who were correctly classified as improved (including patients with a subjective evaluation of “slightly better” and “markedly better”), and specificity was defined as the proportion of patients correctly classified as unimproved (including patients with a subjective evaluation of “no change”, “slightly worse” and “markedly worse”). The area under the curve (AUC) can be interpreted as the probability of correctly discriminating between improved and unimproved individuals and ranges from 0.50 (the same as chance) to 1.0 (perfect discrimination). An initial ROC curve was calculated for the change in NDS scores between the initial assessment (*T*_0_) and the first one after initiation of treatment (*T*_1_), aiming to evaluate the responsiveness in detecting improvement (as this would be expected for most dogs); for this, the subjective scores were grouped as improved and not improved. A second ROC curve was calculated to evaluate responsiveness over time. This compared the NDS score at the first assessment after initiation of treatment (*T*_1_) and a third assessment time (*T*_2_) where the score changed further, either increasing or decreasing (in the cases with clinical suspicion of relapsed, the NDS score of that examination was used here). In cases where no further changes in the NDS score occurred since *T*_1_, the last available assessment was used.

Using correlation analysis, the change score obtained for each NDS score was correlated with the external criterion evaluating change (the subjective clinical evaluation of the response to treatment). As both scales are ordinal data, the Gamma correlation coefficient was used. Larger correlation coefficients indicate a stronger relationship between the results of the outcome measure and the external criterion (Lehman & Velozo, 2010). Correlation coefficients <0.25 are considered as little or no relationship, 0.25 to 0.50 fair relationship, 0.50 to 0.75 moderate‐to‐good relationship and >0.75 are considered good to excellent relationship (Lehman & Velozo, 2010).

## RESULTS

A total of 38 dogs were included in this study. The median age at the time of diagnosis was 60 months (IQR 37 to 85), and there were 22 females (12 neutered) and 16 males (11 neutered). The most common dogs presented were crossbreeds (*n* = 9) followed by the Chihuahua (*n* = 8), French bulldog (*n* = 7), miniature schnauzer, Pug and Yorkshire terrier (*n* = 2 of each), and there was one of each of the following breeds: English bulldog, Boston terrier, cocker spaniel, golden retriever, Jack Russell terrier, Maltese, shih‐tzu and Italian spinone.

The median NDS scores at the different assessments are summarised in Table 1. Median total follow‐up time was 10 months (IQR 6 to 15). Median time at which *T*_1_ was undertaken was 3 weeks after *T*_1_ (IQR 3 to 4 weeks), and median time at which *T*_2_ was undertaken was 6 months after *T*_1_ (IQR 4 to 9 months). At *T*_1_, 35/38 had shown clinical improvement (Table 1), 2/38 remained unchanged, and 1/38 dog showed deterioration. Two dogs did not have an assessment at *T*_2_. One dog was euthanased shortly after *T*_1_ as, despite mild clinical improvement, the owners believed that quality of life was still poor due to ongoing behaviour and mentation changes. The second dog had remained clinically unchanged despite two intravenous administrations of cytosine arabinoside 3 weeks apart and immunosuppressive doses of corticosteroids, so the owners elected to continue a tapering protocol of prednisolone without further assessments; a follow‐up telephone conversation 6 months after diagnosis revealed stable clinical status (mild residual ataxia and tetraparesis).

**Table 1:** Neurodisability scale and subjective clinical evaluation scores at the different assessment times

	*T*_0_ *	*T*_1_ ^†^	*T*_2_ ^‡^
NDS median (IQR)	4 (3 to 7.25)	1 (0 to 2.25)	1 (0 to 3)
Subjective clinical evaluation (*n*, %)			
Markedly worse		0	5 (13.9%)
Slightly worse		1 (2.6%)	8 (22.2%)
No change		4 (10.5%)	14 (38.9%)
Slightly better		12 (31.6%)	8 (22.2%)
Markedly better		21 (65.3%)	1 (2.8%)

* *T*_0_ – initial assessment

^†^ *T*_1_ – first assessment after initiation of treatment

^‡^ *T*_2_ – third assessment time where the score changed further (if this did not occur, the last assessment was used)

**FIG 1:**
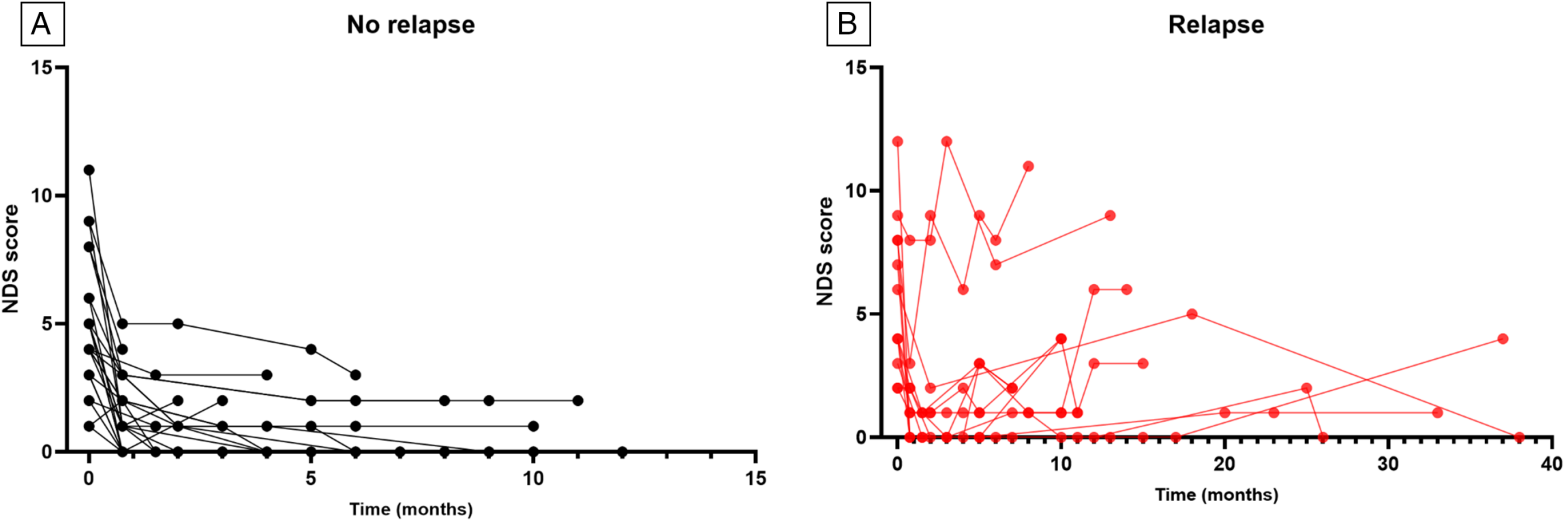
Line graph representing the neurodisability scale (NDS) scores of all dogs. (A) NDS scores of dogs that did not show clinical relapse during the follow‐up period. (B) NDS scores of dogs that were suspected of clinical relapse at different timepoints.

A total of 13 dogs were suspected of clinical relapse during the follow‐up (Fig 1), and this occurred at a median time of 9 months (IQR 5 to 19). Three dogs were euthanased during the study period: one approximately 6 weeks after diagnosis due to insufficient improvement, one 6 months after diagnosis due to suspected relapse and development of diabetes mellitus and one 13 months after diagnosis due to slow progression of the clinical signs (this dog showed some improvement initially but remained with persistent neurological deficits which eventually worsened over time). None of these dogs had *post mortem* examination to confirm the diagnosis.

The NDS achieved excellent responsiveness (Table 2) at both timepoints assessed, with an AUC of the ROC curves of 0.96 at *T*_0_ − *T*_1_ and 0.93 at *T*_1_ – *T*_2_. Furthermore, there was an excellent negative correlation coefficient produced by the change in score between *T*_0_ and *T*_1_ and the dogs’ subjective clinical evaluation (Gamma = −0.8), as well as in the change in score between *T*_1_ and *T*_2_ (Gamma = −0.88).

**Table 2:** Responsiveness of the neurodisability scale assessed by the receiver operating characteristic method and correlation analysis

	AUC (95% CI)	Gamma* coefficient (P value)
Timepoint 1^†^	0.96 (0.89 to 1)	−0.8 (P ≤ 0.001)
Timepoint 2^‡^	0.93 (0.85 to 1)	−0.88 (P ≤ 0.001)

* Correlation between the change in the NDS score and the subjective clinical evaluation of the response to treatment

^†^ This represents the responsiveness evaluation between *T*_0_ (initial assessment) and *T*_1_ (first assessment after initiation of treatment)

^‡^ This represents the responsiveness evaluation between *T*_1_ (first assessment after initiation of treatment) and *T*_2_ (third assessment time where the score changed further; if this did not occur, the last assessment was used)

In seven dogs, the changes in the NDS did not reflect the subjective score. These included six dogs categorised as “no change” that had either decreased one point in the NDS (4/6) or increased one point (2/6). The remaining dog had the same NDS score but was considered “markedly worse”; this dog remained with gait abnormalities (ataxia and paresis) after an initial improvement of the intracranial signs and eventually had a suspected clinical relapse with progression of subtle ataxia to marked ambulatory paraparesis. One dog where the change in the NDS agreed with the subjective score (suggesting relapse) showed newly developed ataxia and obtundation that were secondary to phenobarbital toxicity (also showing severe hepatotoxicity on biochemistry). These resolved with discontinuation of this medication and no other changes to the treatment protocol.

## DISCUSSION

This is the first study to evaluate the responsiveness of the NDS for MUO in dogs, and the results suggest that this scale is responsive to interventions, as well as change over time, suggesting it may be a useful tool in future trials. The NDS was devised to grade clinical severity in patients with MUO and showed good reliability when used prospectively (Gonçalves et al., 2023). This scale is easy to administer and mostly relies on findings of the neurological examination and clinical history, which are always collected during examination of dogs with neurological dysfunction, so it does not require additional interventions. The NDS is calculated by attributing a numerical rating of dysfunction (0 to 3, with the higher number denoting more dysfunction) for the following categories: cerebral functions, cerebellar functions, brainstem functions, visual functions, ambulatory status, postural abnormalities and seizures. It results in an overall score of between 0 (normal) and a theoretical maximum of 21 and provides information about the different types of dysfunction most commonly seen in dogs with MUO (Gonçalves et al., 2023).

To assess responsiveness, criteria that allow identification of patient change (improvement or deterioration) over time is necessary. We used an anchor‐based approach to responsiveness determination, assigning patients to subgroups based on the degree of change in the NDS score compared with external evidence of change, which in this study was the subjective clinical evaluation of the response to treatment. Anchor‐based methods include ROC curves and correlation analysis, and in this study, the results from both methods were in agreement (Narayanaswami, 2017; Revicki et al., 2008). As the purpose of responsiveness studies was to evaluate the measurement property of a given outcome measure and not the amount of therapy effectiveness, there is no need to control the treatment variables for the patients enrolled. In this study, several treatment protocols were used, mostly based on owner preference, and this would have likely affected the overall patient outcomes but have no effect on the ability to evaluate the responsiveness of the NDS. On the other hand, the purpose of clinical trials is to evaluate response to treatment. The availability of psychometrically sound outcome measures is a key issue for clinical trials, with responsiveness to change in major relevance to demonstrate the usefulness of any scale. In most cases, veterinary neurology clinical trials evaluate outcomes such as survival, whilst in human medicine, these focus on outcomes that cannot be directly measured, such as disability, cognitive function or change in symptoms of the condition under study. These more complex outcomes are often subjective and are commonly measured using rating scales. Functional rating scales typically assess the ability of patients to perform tasks and roles for everyday life. The use of composite endpoints, combining multiple clinical outcomes, is also commonly used to more fully capture the multidimensional aspects of chronic diseases; these can include, for example, clinical relapse, changes in disability and MRI activity in neurological disease (Marrie et al., 2023). Using the same COM (such as the NDS) in different studies of MUO may overcome the limitations of survival as an outcome measure and could allow for easier comparison of results, either in isolation or combined with more objective measures such as MRI or other biomarkers.

Care should be taken when using the NDS to monitor patients with significant spinal involvement as highlighted by the dog that showed marked deterioration in the gait, and this was not reflected in the NDS score. The NDS was designed for patients with meningoencephalitis and not meningomyelitis, but many dogs have both. Our results suggest that in dogs with any degree of spinal cord involvement, the NDS should not be used or should be performed alongside spinal cord injury scales (Levine et al., 2009; Olby et al., 2001) to fully evaluate the degree of dysfunction. Conversely, in one dog where both the NDS score and the subjective clinical assessment suggested relapse, the clinical signs were suspected to be secondary to phenobarbital toxicity. It should therefore also be highlighted that deterioration in the NDS score can occur for reasons other than relapse, and these should be thoroughly investigated whenever indicated. Another concern is the category of the NDS related to the occurrence and frequency of seizures. It is possible that patients that are stable or improving show an increase in seizure activity. In such cases, it is difficult to determine whether this is secondary to disease progression or simply postencephalitic epilepsy, and this could increase the NDS score without an actual deterioration in the patient condition. Seizures are a very common presentation in MUO (Bateman & Parent, 1999; Coates et al., 2007; Cornelis et al., 2016; Gonçalves et al., 2024b), so it is very important to document this clinical sign in an outcome measure for this condition, although these changes should be monitored over time and not immediately be considered associated with relapse.

Several limitations should be considered when interpreting the results of this study. The main limitation is the small study cohort, and future studies, with larger populations and including dogs with varying degrees of disability, are required to support our initial results. Due to the need to repeat the assessment at a second timepoint after initiation of treatment, dogs that died early in the disease process or that were lost to follow‐up in the initial weeks were not included. This cohort of patients likely presented more severe signs (and therefore a higher NDS score than our population), so the responsiveness may differ in these patients. This could also be associated with the good outcome seen in most dogs in this study, but these results represent the population that the NDS would most likely be used in, namely those that do not die early in the treatment process and for which treatment trials could be designed. Cases were seen by a board‐certified neurologist or a resident who was supervised by a board‐certified neurologist. The patient was evaluated during the consultation based on clinical and neurological examinations and discussion with the owners – all of this contributed to their subjective clinical assessment, which was then written into the patient’s notes. The NDS score was typically calculated after the consultation by either the neurologist or the resident, and this is therefore a potential source of bias. Ideally, an additional neurological assessment could have been conducted and used to calculate the NDS by a different member of the neurology team; however, this was not practical from an operational point of view. It would have added undue stress to the patient, and we have previously shown that the NDS shows excellent interobserver reliability (Gonçalves et al., 2023). Ideally, the inter‐rater reliability of the subjective clinical assessment should have been determined to evaluate the possible bias introduced by different clinicians, but this would have required two separate patient examinations and owner discussions at each visit, which was not feasible in a longitudinal study such as this. Lastly, it is important to point out that all NDS scores were assigned by neurologists, so the results of this study would likely not apply to general practitioners using this scale. Nonetheless, the NDS is based on findings of the neurological examination, which all veterinarians should know how to perform. Also, when the NDS was designed (Gonçalves et al., 2023), categories that showed poor interobserver reliability (using raters of different experience levels, including rotating interns) were excluded; therefore, the scale should remain useful in different settings, although it will most likely be used by specialists, as these would be the ones monitoring clinical trials.

In conclusion, this study showed that the NDS, previously reported to be a reliable tool to assess dogs with MUO, is also a responsive measure to monitor this condition. This suggests that this scale could be used in clinical trials as a measure of outcome.

## Author contributions

**R. Gonçalves:** Conceptualization; investigation; methodology; writing – original draft; formal analysis; writing – review and editing; data curation. **T. W. Maddox:** Methodology; writing – review and editing; formal analysis; supervision. **S. Phillipps:** Writing – review and editing; data curation. **J. C. Carrete:** Data curation; writing – review and editing. **F. E. Anderson:** Writing – review and editing; data curation. **R. T. Bentley:** Data curation; writing – review and editing. **G. Walmsley:** Conceptualization; writing – review and editing; formal analysis; data curation; supervision.

## Conflict of interest

No conflict of interest has been declared.

## Data Availability

The data that support the findings of this study are available from the corresponding author upon reasonable request.
